# Counseling Role of Primary Care Physicians in Preventing Early Childhood Caries in Children with Congenital Heart Disease

**DOI:** 10.3390/ijerph111212716

**Published:** 2014-12-09

**Authors:** Zifeng Liu, Dongsheng Yu, Lijie Zhou, Jing Yang, Jiaxuan Lu, Hui Lu, Wei Zhao

**Affiliations:** 1 Faculty of Medical Statistics and Epidemiology, School of Public Health, Sun Yat-sen University, Guangzhou 510275, China; E-Mail: sumsliu@hotmail.com; 2 Department of Pediatric Dentistry, Guanghua School of Stomotology, Hospital of Stomatology, Sun Yat-sen University, Guangdong Provincial Key Laboratory of Stomatology, Guangzhou 510275, China; E-Mails: yudsh@mail.sysu.edu.cn (D.Y.); zhoulj7@mail2.sysu.edu.cn (L.Z.); yang_jing1989@126.com (J.Y.); lujiaxuan1989@126.com (J.L.); luhui7@mail2.sysu.edu.cn (H.L.)

**Keywords:** primary care physician, counseling role, early childhood caries, congenital heart disease

## Abstract

The dental health of preschool children with congenital heart disease (CHD) is usually poor, which may contribute to the development of infective endocarditis (IE). Primary care physicians play an important role in providing access to preventive dental services, particularly for preschool children. The object of this study was to provide epidemiologic evidence for the impact of primary care physicians’ (PCP’s) counseling role on early childhood caries in children with CHD in Guangzhou, China, which might guide future caries prevention to decrease the risk of IE in children with CHD. A hospital-based, case-control study was performed, which contained 100 children with newly diagnosed early childhood caries and 100 matched (sex and age) children without dental caries. All of the subjects were diagnosed with CHD at birth and recruited from Guangdong Cardiovascular Institute from 2012 through 2013. A conditional multivariate logistic-regression model was used to assess the associations between PCPs’ role and early childhood caries with a significance level of 5%. Our findings revealed that mother’s education level (OR = 0.36, CL = 0.14–0.92) and knowledge, being educated on the relationship between CHD and infective endocarditis (OR = 0.48, CL = 0.25–0.94) and the impact of oral health on infective endocarditis (OR = 0.37, CL = 0.18–0.79) by the PCP were associated with early childhood caries. PCPs played an important role in preventing early childhood caries among preschool children with CHD in Guangzhou, China.

## 1. Introduction

Congenital heart disease (CHD) is one of the most common types of birth defects, with a mean incidence of approximately 8–10 cases per 1,000 live births [1,2]. Some research has shown that CHD represents a predisposing cardiac condition for the development of infective endocarditis (IE) [3,4], approximately 15% of which was caused by oral organisms and occurred after a recent dental treatment [5,6]. *Streptococcus viridans* from the oral cavity is considered to be the common pathogenic microorganism responsible for IE [2,7], particularly in patients with predisposing cardiac conditions [8]. Therefore, dental health in children with CHD must have more attention paid to it. Primary care physicians (PCPs), as community-based primary care medical providers, play an important role in providing access to preventive dental services, particularly for preschool children. According to the previous research, screening, referral and counseling were the traditionally recommended interventions for the PCP to prevent dental caries in preschool children in some developed countries [9]. On the basis of the results of the screening, the PCP would refer the child to a dentist (identification of suspected caries lesions) or would undertake counseling for reasons that included health promotion and parental education (caries free) [9]. However, for the lack of screening equipment and an oral referral system in most Chinese community clinics, the counseling role of PCPs in preventing dental caries is particularly important.

China is currently experiencing a high prevalence rate of dental caries in five-year-old children (66.0% decayed, missing and filled teeth/dmft = 3.5) [10] and a rapid increase in the incidence rate of CHD (2005 2.396%, 2011 2.882% and 2012 4.095%) [11]. According to the National Prevention and Cure Report on Birth Defects in 2012, there are almost one million newly-diagnosed birth defects in children per year, about 26.7% of which are CHD, which ranks first [11]. Being influenced by the Chinese traditional culture, such as the old saying ‘a toothache is not a disease actually’, parents habitually considered dental health as a low priority in the grander scheme of birth defects. PCPs see most preschool children at least once a year (in the community clinics or kindergarten) for physical examination in many communities in Guangzhou. Thus, strengthening PCP’s caries preventive role in children with CHD and improving parental knowledge of dental health are very important. However, until now, few studies have been conducted to evaluate the caries preventive effectiveness of PCPs’ role in children with CHD, which goes against the work of caries prevention. Therefore, we performed this case-control study to provide exploratory epidemiologic evidence for the PCPs’ counseling role in preventing early childhood caries (ECC), which might guide future caries prevention to decrease the risk of IE in children with CHD.

## 2. Method

### 2.1. Study Design and Patients

We performed a hospital-based, case-control study of 100 children with newly-diagnosed ECC (case group) and 100 children without dental caries (control group) to evaluate the impact of PCPs’ counseling role on ECC in children with CHD. The children in this study were recruited from Guangdong Cardiovascular Institute in Guangzhou from 2012 through 2013, all of whom had been diagnosed with CHD by the doctors based on MRI or multi-slice CT at birth. They received child care at least once a year by a PCP. Eligible cases included children with CHD with a confirmed diagnosis of ECC. The control group consisted of children with CHD without dental caries. Subsequent to enrollment to a case, eligible control children within the same sex and 6-month-old categories were utilized until one control patient was matched to each case patient. The parents of each child were informed of the study design and the dental examination proposed for their children. All of the parents in both groups voluntarily joined this study with written informed consents.

Ethical approval was obtained from the Ethical Review Committee of the Guanghua School of Stomotology, Hospital of Stomatology, and Sun Yat-sen University (ERC-2011-036). Written informed consent for participation was obtained from the parents on behalf of their children enrolled in our study.

### 2.2. Questionnaires

After reviewing the relevant literature, socio-demographic characteristics, socio-economic conditions and 8 important questions on the effectiveness of PCPs’ counseling role were asked to evaluate whether the PCP had told the parents the following information: (1) the oral health impacts on the health of the body; (2) that children with CHD are at risk of IE; (3) the dental caries is related to IE; (4) that brushing teeth can prevent decay; (5) that eating and drinking sweet food can cause decay; (6) that flossing teeth can prevent tooth decay; (7) that the use of fluoride prevents tooth decay; and (8) that regular dental visits are necessary. Questionnaires containing these 8 questions were used for interviewing parents by trained interviewers who were medical students at Sun Yat-Sen University. In order to reduce the recall bias, we allowed enough time for parents to recall what the PCPs had told them.

### 2.3. Dental Caries Examination

The clinical exam and diagnosis of ECC was performed by two experienced dentists who had undergone a calibration procedure (kappa = 0.90). The evaluations were carried out with a dental probe and a mouth mirror under artificial white light, according to the criteria established by the WHO for caries in primary dentition [12].

### 2.4. Statistical Analysis

Data processing and analysis were performed by SPSS 13.0 for Windows, with the level of significance set at 5.0% (*p*-value < 0.05). Percentages, means and standard deviations (SD) were computed for descriptive purposes. A conditional multivariate logistic-regression model was used to assess associations by the adjusted odds ratio (OR) with a 95% confidence interval (95% CI) between dental caries status and PCPs’ role. Caries prevalence, as a dependent variable, was categorized into a dichotomous variable: ECC and caries free. The independent variables were the 8 questions on the role of PCP answered as “yes” and “no”. Potential confounding factors were socio-demographic characteristics (parental education level, domestic economic status and the number of child in the family).

## 3. Result

### 3.1. Description of the Study Sample

We enrolled 113 consecutive patients with newly-diagnosed ECC from 2012 through 2013 in order to reduce the selection bias, and 100 patients (88.5%) agreed to participate in our study. The same number of control patients was enrolled. Both groups were matched in terms of age and gender. The average age of the patients was 4.7 years (SD = 1.2), and 56.0% were male.

### 3.2. Single-Factor Analysis

In the conditional univariate logistic-regression analysis (Table 1), case and control children were similar with regard to gender, father’s education level, domestic economic status and number of child in the family, but children whose mother’s education level was higher would have a lower risk of ECC (crude OR = 0.30, CI = 0.12–0.75). Table 2 shows the results of single-factor analysis for the relationship between PCPs’ counseling role and ECC in children with CHD. If the PCPs had told the parents that children with CHD are at risk of IE (crude OR = 0.42, CI = 0.23–0.79) and dental caries is related to IE (crude OR = 0.32, CI = 0.16–0.66), the children would have a lower risk of ECC.

### 3.3. Conditional Multivariate Logistic-Regression Analysis

Table 3 shows results from a conditional multivariate logistic regression model, which identified the PCPs’ counseling role. Preschool children were more likely to have a lower risk of ECC if their mothers’ education level was high (OR = 0.36, CI = 0.14–0.92), their parents were told that children with CHD are at risk of IE (OR = 0.48, CI = 0.25–0.94) and that dental caries is related to IE (OR = 0.37, CI = 0.18–0.79).

**Table 1:** Odds ratio for conditional logistic regression between social-demographic characteristics and caries experience in children with CHD.

Characteristics	Case Group (N = 100) (number (percent))	Control Group (N = 100) (number (percent))	Unadjusted OR (95% CI) ^b^		*p ^a^*
*Gender*					
Male	56 (50.0)	56 (50.0)	---	---	---
Female	44 (50.0)	44 (50.0)	---	---	
*Father’s education level*					
>12 year	20 (55.6)	16 (44.4)	1.44	(0.62–3.78)	0.40
≤12 year	80 (48.8)	84 (51.2)	1.00		
*Mother’s education level*					
>12 year	8 (26.7)	23 (74.2)	0.30	(0.12–0.75)	<0.01
≤12 year	92 (54.1)	77 (45.6)	1.00		
*Domestic economic status*					
High-income	8 (53.3)	7 (46.7)	1.10	(0.59–2.05)	0.77
Middle-income	69 (50.4)	68 (49.6)	1.26	(0.38–4.24)	0.71
Low-income	23 (47.9)	25 (52.1)	1.00		
*Only-child*					
Yes	69 (50.4)	68 (49.6)	0.94	(0.49–1.83)	0.87
No	31 (49.2)	32 (50.8)	1.00		

^a^ Conditional univariate logistic-regression; *p* < 0.05 is considered statistically significant. ^b^ OR (95% CI) = odds ratio (95% confidence interval).

**Table 2:** Odds ratio for conditional logistic regression between parental knowledge from primary care physicians (PCPs) and caries experience in children with CHD. IE, infective endocarditis.

Variables	Case group (N = 100) (number (percent))	Control group (N = 100) (number (percent))		Unadjusted OR (95% CI) ^b^		*p ^a^*
*Oral health impacts the health of the body*						
Yes	28 (53.8)	24 (46.2)	1.27		(0.64–2.49)	0.49
No	72 (48.6)	76 (51.4)	1.00			
*Children with CHD are at risk of IE*						
Yes	35 (39.3)	54 (60.7)	0.42		(0.23–0.79)	<0.01
No	65 (58.6)	46 (41.4)	1.00			
*Dental caries is related to IE*						
Yes	13 (27.7)	34 (72.3)	0.32		(0.16–0.66)	<0.01
No	87 (56.9)	66 (43.1)	1.00			
*Brushing teeth can prevent decay*						
Yes	65 (49.6)	66 (50.4)	0.93		(0.45–1.93)	0.85
No	35 (50.7)	34 (49.3)	1.00			
*Regular dental visits are necessary*						
Yes	10 (43.5)	13 (56.5)	0.67		(0.24–1.88)	0.44
No	90 (50.8)	87 (49.2)	1.00			
*Flossing teeth can prevent tooth decay*						
Yes	22 (53.7)	19 (46.3)	1.23		(0.59–2.56)	0.58
No	78 (49.1)	81 (50.9)	1.00			
*Eating and drinking sweet food can cause decay*						
Yes	90 (50.6)	88 (49.4)	1.25		(0.49–3.17)	0.64
No	10 (45.5)	12 (54.5)	1.00			
*Use of fluoride prevents tooth decay*						
Yes	19 (41.3)	27 (58.7)	0.53		(0.24–1.19)	0.12
No	81 (52.6)	73 (47.4)	1.00			

^a^ Conditional univariate logistic-regression; *p* < 0.05 is considered statistically significant. ^b^ OR (95% CI) = odds ratio (95% confidence interval).

**Table 3:** Odds ratio for the results of the conditional multivariate logistic regression between independent variables and caries experience in children with CHD.

Variables	Adjusted OR (95% CI) ^b^		*p ^a^*
*Mother’s education level*			
High	0.36	(0.14–0.92)	0.03
Low	1.00		
*Children with CHD are at risk of IE*			
Yes	0.48	(0.25–0.94)	0.03
No	1.00		
*Dental caries is related to IE*			
Yes	0.37	(0.18–0.79)	0.01
No	1.00		

^a^ Conditional multivariate logistic-regression; *p* < 0.05 is considered statistically significant. ^b^ OR (95% CI) = odds ratio (95% confidence interval).

## 4. Discussion

Even though it is widely known that good oral health in preventing bacteremia and IE is very important [13,14], the majority of studies with heart disease children report that the dental health of these subjects is usually poor [15,16]. The early and frequent contact that most preschool children typically have with PCPs presents a unique opportunity to evaluate their oral condition and perform basic preventive services. This hospital-based, case-control study investigated the roles of PCPs regarding ECC in the primary teeth among preschool children in Guangzhou, China. It confirms the important counseling roles of PCPs for the prevention of ECC in children with CHD. Some confounding variables, especially socio-demographic characteristics and parental education level, were also taken into account in this study. According to the results of conditional multivariate logistic-regression analysis, three variables were associated with the development of ECC in children with CHD (*p* < 0.05). These were mother’s educational level, her education about the relationship between CHD and IE and the impact of oral health on IE.

Mother’s educational level was related to the risk of ECC, which was supported by other researchers in different countries [17,18,19,20]. This can be explained by low educational level being associated with reduced ability to adapt to health promoting behavior [20] and by mothers with low education not initiating oral hygiene practices in their preschool child in a timely manner [21]. In China, children mostly were taken care of by the mother, whose poor feeding practices, such as breast-feeding during the nighttime and irregular tooth brushing [22,23], could lead to the development of ECC in children [24]. On the other hand, most families have only one child (85.0%, case group; 80.0% control group) in the preset study. The mothers spoiled the children and found it harder to implement supervision into action, which also might contribute to the development of ECC. Besides, mothers with a higher educational level were more likely to actively acquire oral health knowledge from mass media, dentists or PCPs, which might decrease the likelihood of ECC.

In addition, parents being educated on the relationship between CHD and IE and the impact of dental caries on IE could significantly affect the children’s oral health status (*p* < 0.01). For children with CHD, their parents habitually considered dental health as a low priority in the grand scheme of birth defects. In China, because of the lack of dentists [25] and insufficient projects for public information and education about oral health, few parents have taken their child for regular dental visits. By not taking their children to a dentist, they lose an important source of knowledge about dental health, and this vicious cycle continues as such. Moreover, according to traditional Chinese concepts, the ‘internal heat’ of the body is considered a cause of some dental diseases. Many Chinese people believe that consumption of some ‘hot’ food will increase the ‘internal heat’, which is the main reason for dental disease, while the drinking of herbal tea, instead of visiting a dentist, is enough. PCPs, as community-based primary care medical providers, may be the earliest and most frequented specialists that the parents of preschool heart disease children can contact. By receiving professional advice from the PCPs, the parents acquired an important source of knowledge about dental health and IE, which might change the parental perception of their children’s dental health. They would pay more attention to their children’s dental health and help him/her to develop a good habit of oral cleaning, which might decrease the prevalence of ECC and the risk of IE consequently.

In this study, only a minority of parents had ever been told the importance of oral health in children with heart disease by the PCPs (13.0% in the case group, 34.0% in the control group), which meant that most PCPs had not realized the importance of dental health in children with CHD or lacked relevant dental knowledge, which may act as a barrier to their dental healthcare delivery. Other researchers also reported a lack of oral health knowledge among doctors in Canada and the USA [26,27]. In China, the general practitioner (GP) system has not been established so far. Most PCPs in community clinics come from graduate programs in clinical medicine, instead of being GPs. They study a few clinical courses of stomatology and are not competent enough to examine children’s dental health. Thus, comprehensive oral health educational programs for PCPs are urgently needed now, and this education can be done by dentists and dental staff [28], which would provide an important basis for enhancing their role in preventing dental caries among children with heart disease.

## 5. Conclusions

In conclusion, this study provided rigorous epidemiologic evidence of the important roles of PCPs in preventing ECC among children with congenital heart disease in Guangzhou, China. Mother’s education level, parental awareness of IE and the impact of oral health on IE were associated with the development of ECC in children with CHD. In addition, education on improving the PCPs’ knowledge and perception of oral health is also imperative now, which may provide an important basis for enhancing their role in preventing dental caries in children with CHD.
